# Efficacy and Safety of Angioplasty Balloon Interposition in CT-Guided Percutaneous Thermal Ablation of Hepatic Malignancies to Protect Adjacent Organs

**DOI:** 10.1007/s00270-022-03184-1

**Published:** 2022-07-06

**Authors:** Michael Schlappa, Wolfgang Wüst, Jürgen Siebler, Robert Grützmann, Michael Uder, Axel Schmid

**Affiliations:** 1 Institute of Radiology, University Hospital, Friedrich-Alexander-University Erlangen-Nürnberg, Erlangen, Germany; 2 Institute of Radiology, Martha-Maria Hospital Nürnberg, Nuremberg, Germany; 3 Department of Medicine 1, University Hospital, Friedrich-Alexander-University Erlangen-Nürnberg, Erlangen, Germany; 4 Department of Surgery, University Hospital, Friedrich-Alexander-University Erlangen-Nürnberg, Erlangen, Germany

**Keywords:** Liver tumour, Thermal ablation, Balloon interposition, Organ protection, Organ displacement

## Abstract

**Purpose:**

To evaluate the feasibility and safety of placing angioplasty balloons between the liver surface and adjacent organs in CT-guided thermal ablation of subcapsular liver malignancies in case of inadequate success of conventional dissection techniques.

**Materials and Methods:**

A retrospective, single-centre database query identified 327 hepatic malignancies in 153 patients treated in 215 sessions from 2016 to 2018 by thermal ablation. Demographic data, tumour size, distance to adjacent structures, complications and long-term outcomes were assessed when ancillary procedures were performed to protect adjacent organs.

**Results:**

In 21 of 327 (6.4%) ablations, thermal protection was necessary. Balloon interposition was successfully performed in 9 cases in 8 patients after hydrodissection or gas insufflation failed to separate adherent organs. Median pre- and post-balloon insertion distance was 0 mm [0–2 mm] and 17 mm [8–20 mm]. No balloons were damaged, ruptured or slid away from their initial position. Technical success of MWA and protection of adherent structures were achieved in all procedures. In a median follow-up of 11.5 months [0–49 months], the local control rate was 88.9% as 1 patient was treated twice with an interval of 3 months for local recurrence. Three non-process-related major complications and 1 minor complication occurred.

**Conclusion:**

Balloon interposition is a safe and feasible method to enable thermal ablation to a greater number of patients, even after established thermo-protective techniques fail to separate the colon or stomach from the liver surface.

## Introduction

Thermal ablation is a well-established, minimally invasive alternative to resection of HCC and liver metastases. It is considered safe with a recent meta-analysis showing minor complications in 5.7% and major complications in 4.6% with a mortality rate of 0.23% [1]. Nevertheless, performing percutaneous ablation near adjacent organs results in the risk of thermal damage, potentially leading to serious complications such as gastrointestinal perforation. Therefore, thermo-protective techniques like gas insufflation, hydrodissection, levering the adherent organ with blunt-tip needles and bile aspiration have been established [2–4]. If foregoing measures fail to displace adherent structures, single case reports imply that the balloon interposition technique may be a feasible second-line procedure for organ protection [5–7]. Relevant case series to evaluate technical feasibility, safety and success do not exist for now. This retrospective, single-centre case series presents nine cases.^1^

## Materials and Methods

### Study Population

From 2016 to 2018, all patients who underwent CT-guided thermal ablations were identified in a retrospective database query. If preceding traditional dissection methods did not achieve to adequately isolate the adherent structures, balloon catheter interposition was performed as second-line treatment at our institution. The study was approved by the institutional review board.

### Balloon Interposition Technique

**Fig. 1:**
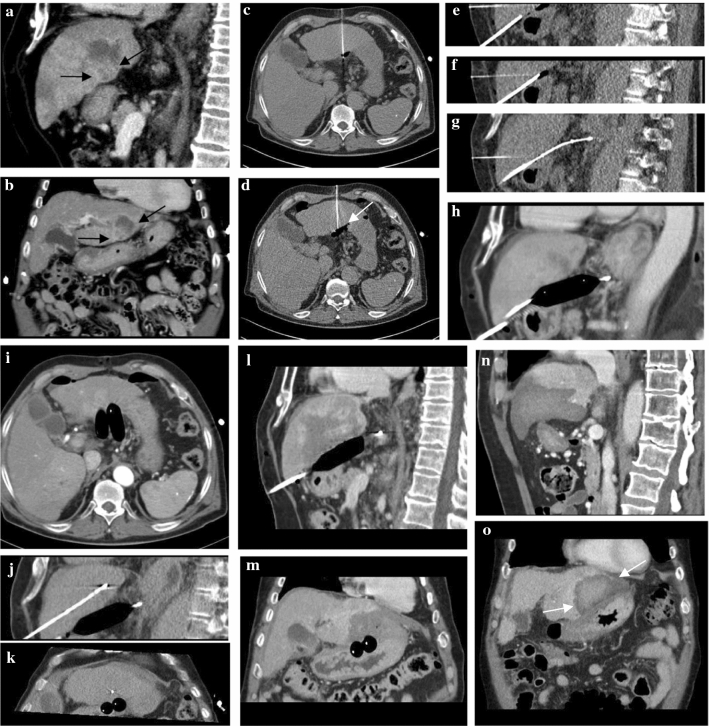
Preinterventional CT scan of a 73y patient with a HCC (black arrows) in S3 adjacent to the stomach (**a**, **b**). Insufficient technical success of gas dissection (white arrow, **c**, **d**). Positioning of guiding needles in between the liver and the stomach by using blunt trocars (**e**, **f**). Advancing angioplasty balloons via 8F sheaths over guiding wires (**g**, **h**, **i**). Placement of the microwave antenna (**j**, **k**). CT scan immediately (**l**, **m**) and 2 days after thermoablation (**n**, **o**) shows the periablational zone covering the entire HCC (white arrows). Complete ablation was confirmed by follow-up. No complication to the stomach occurred

**Fig. 2:**
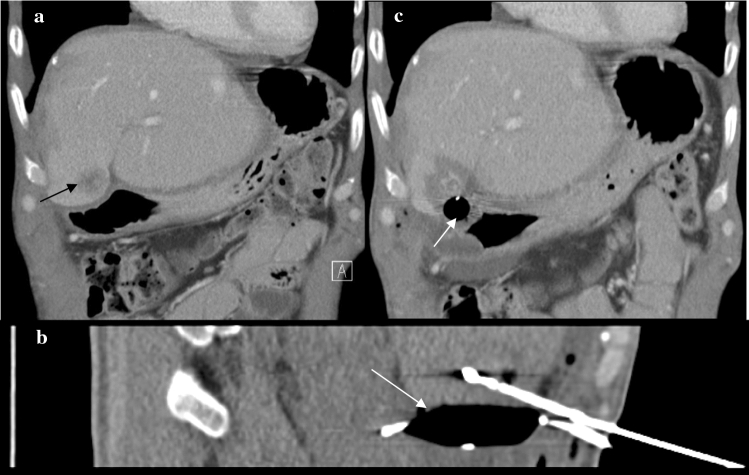
**a** Preinterventional CT-scan of a 56-year old male patient previously treated by right hemihepatectomy with a new liver metastasis (black arrow) from rectal carcinoma in segment IVb adjacent to the stomach. **b** Positioning of the MW antenna following interposition of an angioplasty balloon (white arrow) between the liver and the stomach. **c** Postinterventional CT-scan showing the balloon in between the ablation zone and the stomach wall

**Fig. 3:**
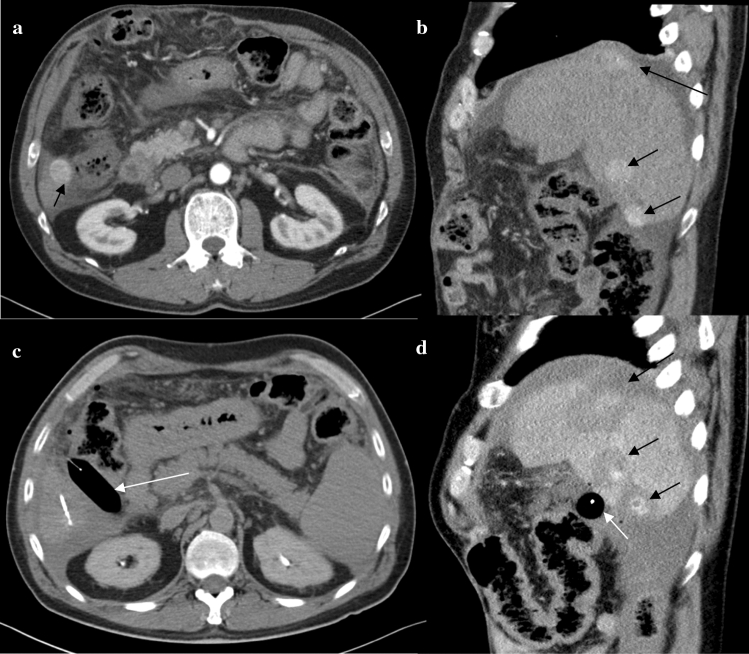
**a**, **b** Preinterventional CT-scan of a 60-year old male patient with liver cirrhosis and multifocal HCC (black arrows). Short distance between HCC tumours in S6 and the right colonic flexure. **c** MW ablation of the lesion in S6 following interposition of an angioplasty balloon (white arrow) between the liver and the colon. **d** Postinterventional CT-scan showing the balloon positioned between the ablation zone and the colon

All procedures were performed under general anaesthesia and with CT guidance by using dedicated software for 3D CT guided interventions (Adaptive 3D Interventional Suite, Siemens, Forchheim, Germany). The abdomen was punctured with a 17-gauge coaxial biopsy needle (TruGuide®, BARD® Peripheral Vascular Inc., Tempe, AZ, USA) which contains an optional blunt-tip stylet. After accessing the peritoneum, the trocar-tip stylet was replaced by the blunt-tip stylet in order to minimize the risk of damage to peritoneal organs when gradually advancing the needle in between the liver surface and adjacent organs [8]. A 0.035 ‘‘J-tip PTFE guiding wire (Emerald®, Cordis, Santa Clara, CA, USA) was then placed 5–10 cm beyond the needle and an 8–9 Fr sheath (Radifocus®, Terumo Corporation, Shibuya, Tokyo, Japan) was advanced over the wire just into the peritoneal cavity. An angioplasty balloon (16/40 mm, 18/40 mm or 20/40 mm, ATLAS® Percutaneous Transluminal Angioplasty Balloons, Bard Peripheral Vascular, Inc., Tempe, AZ, USA) was placed through the sheath and was inflated manually with air with a 5 ml Luer Lock Syringe when in correct position (Figs. 1, 2 and 3). In case of insufficient separation of the adjacent organ from the liver surface, a second angioplasty balloon was placed in parallel to the first balloon by using the same technique. Finally, the MWA antenna was placed in the intended liver position and the ablation including track ablation was performed with standard ablation protocols [9].

### Data Analysis

CT scans, treatment protocols and physician letters were reviewed for demographic data, tumour type, lesion size, liver segment, type of organ in need of protection, pre- and post-balloon insertion distance between liver and adjacent organ, number of balloons used, displacement of balloon, time to insert balloons, technical success, complications and local control rate at last available follow-up. Adequacy of organ protection was judged bases on the distance between liver surface and the adjacent by the interventionalist. Technical success was defined as complete ablation of the lesion plus an ablative margin of 5 mm for HCC and 10 mm for metastases on first follow-up CT imaging 2 days and second follow-up CT or MRI 8 weeks after the procedure. Complications were defined according to the CIRSE classification.

## Results

In 21 of 327 lesions (6.4%) treated from 2016 to 2018, ancillary procedures were necessary to perform thermal ablation (Table 1). Consecutive balloon interposition was performed in 9 cases in 8 patients when preceding gas- or hydrodissection failed to separate adjacent organs from the liver capsule (42.9%). The treated patients suffered from HCC (*n* = 4), and metastases from either CRC (*n* = 3) or oesophageal adenocarcinoma (*n* = 1). Median tumour size was 31.5 mm [21–42 mm]. Six malignancies were located in segment 3, the other 3 tumours sited in segments 2, 4b and 6. In 7 procedures, the stomach was the adjacent organ, while the colon was adherent in 2 cases. Median pre- and post-balloon insertion distance was 0 mm [0–2 mm] and 17 mm [8–20 mm]. In 3 cases, a single balloon was effective for organ protection, and a second balloon was necessary in six cases. Median duration of the complete intervention was 2 h 39 min [1 h 57 min–3 h 50 min]. The time investment of balloon interposition itself was retrospectively evaluable only in the first 2 cases (67 and 34 min). Technical success of MWA was achieved in all cases. No ablations had to be aborted because of inadequate organ protection. No balloon was damaged, ruptured, or slid away from its initial position throughout the ablation. No thermal damage to protected organs was observed. No complications were caused by the placement, insufflation or removal of the balloons. Three non-process-related major complications occurred (Table 2). One patient was treated twice in an interval of 3 months for local recurrence. In a median follow-up of 11.5 months [0–49 months], no local recurrences occurred in the other patients. No complications caused by the placement or removal of the balloons occurred.

**Table 1:** A53 thermal ablations with organ protection from 2016 to 2018

Variables	No. of ablations
Thermal ablation with organ protection	21/327 (6.4%)
Hydrodissection	17/21 (80.0%)
Gas- and hydrodissection	1/21 (4.8%)
Bile aspiration	3/21 (14.3%)
Consecutive balloon interposition	9/21 (42.9%)
Technical success	21/21 (100%)
2016–2018 without balloon interposition	12/21 (57.1%)
2016–2018 after consecutive balloon interposition	21/21 (100%)

**Table 2:** Thermal ablations of liver lesions close to the liver capsule enabled by balloon interposition after initial failure of gas-/hydrodissection

Pat	Age	Sex	Entity	Seg	Tumour size (mm)	PTA catheters	Distance to adherent organ pre and post balloon insertion (mm)	Thermal ablation	Primary dissection technique	Time to deploy balloon/total time of the intervention	Complications to the protected organ	Other complications	Local recurrence and follow-up (months)
1	73	M	HCC	3	34 × 41 × 40	2 balloons (20/40 mm, 18/40 mm)	Pre insertion: 0 Post insertion: 20	MWA—5 ablations	Hydro- and gas- dissection	1 h 7 min/3 h 27 min	No—Stomach	Renal forniceal rupture	No (FU 2)
2	68	M	HCC	3	31 × 24 × 23	2 balloons (16/40 mm)	Pre insertion: 0 Post insertion: 8	MWA—3 ablations	Hydrodissection	34 min/2 h 7 min	No—Stomach	Brachial plexus injury	No (FU 49)
3	56	M	CRC	4b	28 × 27 × 26	1 balloon (16/40 mm)	Pre insertion: 0 Post insertion: 18	MWA—4 ablation	Hydrodissection	N/A/2 h 39 min	No—Colon	No	No (FU 0)
4	60	M	HCC	6	21 × 18 × 21	1 balloon (16/40 mm)	Pre insertion: 0 Post insertion: 17	MWA—3 ablations	Hydrodissection	N/A/3 h 36 min	No—Colon	No	No (FU 13)
5	26	M	HCC	3	32 × 22 × 23	2 balloons (16/40 mm)	Pre insertion: 0 Post insertion: 9	MWA—2 ablations	Hydrodissection	N/A/1 h 57 min	No—Stomach	No	No (FU 1)
6	56	W	CRC	3	22 × 23 × 21	1 balloon (18/40 mm)	Pre insertion: 0 Post insertion: 8	MWA—2 ablations	Hydrodissection	N/A/2 h 2 min	No—Stomach and colon	No	No (FU 23)
7a	83	M	HCC	3	38 × 41 × 47	2 balloons (20/40 mm)	Pre insertion: 0 Post insertion: 17	MWA—5 ablations	Hydrodissection	N/A/3 h 50 min	No—Stomach	Pleural effusion	Yes (after 3)
7b	83	M	HCC	3	41 × 15 × 32	2 balloons (20/40 mm)	Pre insertion: 0 Post insertion: 18	MWA—3ablations	Hydrodissection	N/A/2 h 22 min	No—Stomach	No	No (FU 8)
8	71	M	EAC	3	42 × 39 × 25	2 balloons (16/40 mm, 18/40 mm)	Pre insertion: 2 Post insertion:8	MWA—1 ablation	Hydrodissection	N/A/2 h 57 min	No—Stomach	No	No (FU 17)

Technical success was achieved in all procedures. Patient 1 was prophylactically treated with PPI for 4 weeks. Patient 7 was treated twice with an interval of 3 months for local recurrence.

*N/A* not available. *FU* follow-up

## Discussion

Even though thermal ablation is rated as an effective and safe method, its use is limited by several factors like tumour size or distance between the ablation zone and crucial structures, as they determine both the technical success and the risk of complications, such as bowel perforation [10–12]. As most neoplasms can be separated effectively from the adjacent organ by traditional dissection methods, these methods are considered first-line procedures [7, 9, 13–16,17]. However, their technical success might be limited if post-operative adhesions are present or if the administered gas or fluids disperse away from the intended site [6, 7, 13]. In these cases, balloon interposition seems to emerge as a valuable additional option to finalize complex ablative liver procedures as the given data indicates that 9 of 21 ablations could not have been successfully treated without balloon interposition. Furthermore, it may also help to achieve complete ablation by enabling a more aggressive treatment of the tumour.

**Fig. 4:**
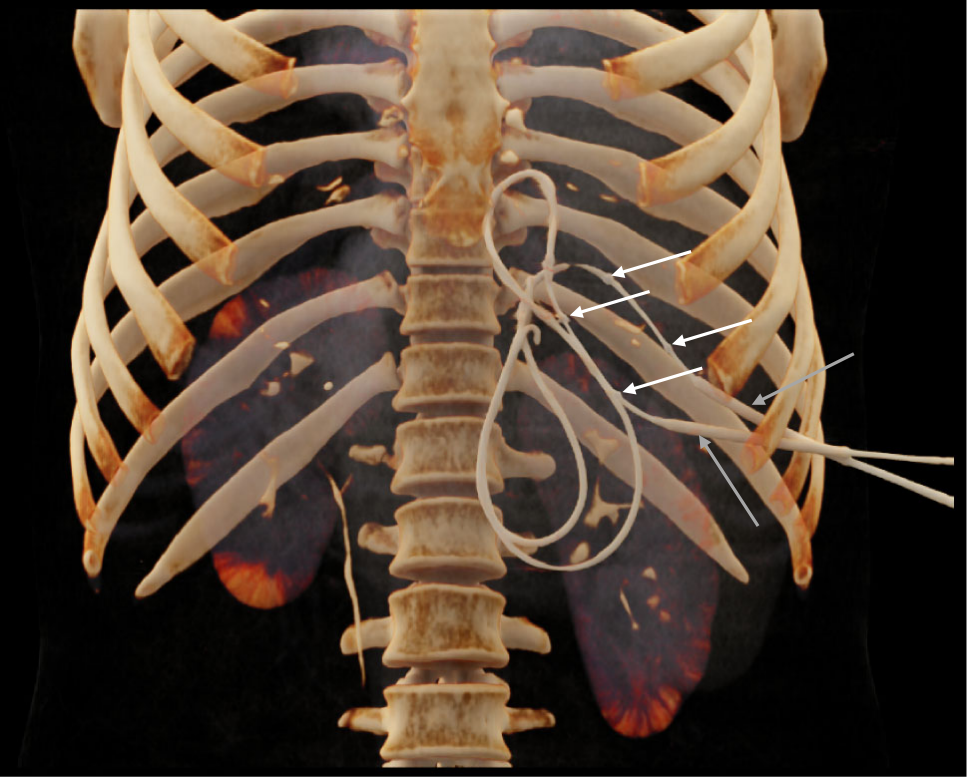
Case example showing cranial deviation of the proximal guiding wires caused by a too deep placement of the wires resulting in a cranial dislocation of the angioplasty balloons (white arrows = balloon markers, gray arrows = guiding sheaths)

So far, only an animal model and single case reports have been published on this technique [5, 18]. The tendency of the balloon to dislocate from its intended position might has been discussed as the main disadvantage of the procedure [7]. In our experience, the displacement of the balloon usually results from advancing the guiding wire too deeply into the peritoneal space leading to contact of the wire tip to peritoneal structures and hence, a lateral movement of the wire body (Fig. 4). Therefore, the dislocation of the balloon could be prevented by advancing the wire only a few centimetres beyond the targeted balloon position.

This study has certain limitations. Firstly, this single-centre case series contains only 9 procedures in which balloon interposition was performed. Hence, to evaluate technical success and safety further in-depth data are needed. Secondly, due to the retrospective approach of this study the duration of balloon interposition itself was only determinable in 2 patients. Even if a learning curve can be assumed balloon interposition will add further time exposure which should be taken into consideration when planning complex ablation procedures.

## Conclusion

In summary, balloon interposition is a feasible, safe and effective second-line technique to protect the colon or stomach during percutaneous thermal ablation of subcapsular hepatic lesions.

## Footnotes

1 The present work was performed in partial fulfillment of the requirements for obtaining the degree „Dr. med.” at Friedrich-Alexander-Universität Erlangen-Nürnberg (FAU).
